# Antifungal activity of potential probiotic *Limosilactobacillus fermentum* strains and their role against toxigenic aflatoxin-producing aspergilli

**DOI:** 10.1038/s41598-023-27721-1

**Published:** 2023-01-08

**Authors:** Yalda Mahjoory, Reza Mohammadi, Mohammad Amin Hejazi, Yousef Nami

**Affiliations:** 1 Department of Soil Science, Faculty of Agriculture, University of Tabriz, Tabriz, Iran; 2 Department of Genomics, Branch for Northwest & West Region, Agricultural Biotechnology Research, Institute of Iran, Agricultural Research, Education and Extension Organization (AREEO), Tabriz, Iran; 3 Department of Food Biotechnology, Branch for Northwest & West Region, Agricultural Biotechnology Research, Institute of Iran, Agricultural Research, Education and Extension Organization (AREEO), Tabriz, Iran

**Keywords:** Biotechnology, Microbiology

## Abstract

Two major aflatoxin-producing strains are *Aspergillus flavus* and *Aspergillus niger*. Probiotic bacteria have been identified as a potential means to fight aspergilli and reduce the availability of aflatoxin (AFs) as well as other food contaminants. In this study, the potential of ABRIIFBI-6 and ABRIIFBI-7 strains to inhibit the growth of aspergilli was investigated. Both strains survived in the simulated gastrointestinal conditions and inhibited the growth of *Aspergillus* significantly. Auto-aggregation ranged from 67.4 ± 1.9 for ABRIIFBI-6 to 75.8 ± 2.3% for ABRIIFBI-7, and hydrophobicity ranged from 57.3 ± 1.6 to 61.2 ± 1.4% for ABRIIFBI-6 and ranged from 51.2 ± 1.4 to 55.4 ± 1.8% for ABRIIFBI-7. The ranges of coaggregation with *Staphylococcus aureus* were 51.3 ± 1.7 and 52.4 ± 1.8% for ABRIIFBI-6 and ABRIIFBI-7, respectively, while coaggregation with Bacillus cereus was 57.9 ± 2.1 and 49.3 ± 1.9% for ABRIIFBI-6 and ABRIIFBI-7, respectively. Both strains indicated remarkable sensitivity to clinical antibiotics. According to the analysis of the identified potential probiotics, the findings of this study could significantly contribute to the understanding of the probiotic potential of LAB in dairy products in order to access their probiotic characterization for use as biocontrol of aflatoxin-producing species.

## Introduction

Moldy contamination of basic foods like cereal has been identified as one of the most important food safety issues. This is owing to the acute and chronic harm they cause to people and animals. Because of their biological and metabolic impacts on living systems, *Aspergillus flavus* and *Aspergillus niger* are very significant^1^. As secondary metabolites, *A. flavus* and *A. niger*, as well as other *Aspergillus* species, produce aflatoxins^2^. Molds that produce aflatoxin are found all over the world and can thrive on a variety of food and feed commodities during production, processing, storage, and transportation^3^. Aflatoxin production in food is controlled by a variety of factors, including temperature and humidity^4^. When these molds contaminate crops, particularly in hot and humid conditions, customers incur health issues. Aflatoxin is a potent carcinogen and mutagen with hepatotoxic and immunosuppressive properties, as well as the ability to disrupt various metabolic activities, resulting in liver and kidney damage^5^. B1, B2, G1, and G2 are the four most frequent aflatoxins discovered in mold-infested food crops. When nursing animals consume aflatoxin B1 (AFB1) via feed, it is physically changed in the body and released into the milk as aflatoxin M1 (AFM1)^6^. Furthermore, since aflatoxin is not digested by animals, its remains may be found in their meat^7^. Aflatoxin is one of the most dangerous food pollutants because even low doses are mutagenic and can cause hepatotoxicity and teratogenicity^8^. AFB1 is the most dangerous of the four primary forms of aflatoxins, and it has been classified as a human carcinogen by the International Agency for Research on Cancer (IARC) because it causes hepatic cancer^9^.

Government bodies all throughout the globe monitor the level of aflatoxin contamination in food^8^. The maximum allowed level for AFB1 in the European Union is 2 ppb; in the United States, it is 20 ppb^10^. Because of AFB1’s high thermal and mechanical stability, detoxification of contaminated food products is difficult. The concentration of aflatoxin in cereal grains is unaffected by processing^11^. There are various physical and chemical methods to prevent fungal and aflatoxin contamination, but these methods are non-biodegradable and must be applied to the product via heat, ionizing radiation, or pesticide and fungicide spraying. However, it causes both sensory changes and irreversible environmental damage^12^.

As a result, an effective, ecologically acceptable, and customizable approach for preventing aflatoxin contamination of food crops is required. Biological control, which includes the use of bacterial probiotic strains, has shown to be an effective method of achieving the same outcomes. Probiotics are among the nutritional supplements with the highest worldwide growth. Their commercial success in food is much greater. Yogurt is the most common source of probiotics, but many other foods, including as cheese, fruit juices, and cereals, are becoming more popular^13,14^.

Probiotic microorganisms, according to FAO/WHO, are “living microorganisms that, when consumed in appropriate proportions, provide health benefits to the host”^15–17^. This definition highlights the core of probiotics while addressing a broad variety of bacteria and uses. The definition distinguishes between live microbes used as processing aids or sources of useful compounds and those administered primarily for their health benefits. Although commensals in the gut are frequently the source of probiotic strains, they cannot be called probiotics until these strains are isolated and characterized and a credible case for their health effects is presented. As a result, the term “probiotic” has been restricted in some countries because it is deemed misleading to consumers in the absence of approved health claims^17^.

Many grains have long been fermented with probiotic bacteria to increase their nutritional content while also acting as antimicrobial agents, making them safer to consume. The production of organic acids and bacteriocins by probiotic lactic acid bacteria (LAB) has been linked to antibacterial activity^18^. Some of these LABs, particularly those belonging to the genus *Lactobacillus*, have been shown to inhibit fungal growth. Antifungal compounds such as phenyl lactic acid, p-hydroxyl phenyl lactic acid, and other antifungal cyclic dipeptides are produced as a result^19^. This property is provided by the various antifungal chemicals produced primarily at the beginning of the process. In addition to its antifungal properties, LAB has been shown to bind and eliminate aflatoxin in vitro and in vivo^18^.

Probiotics have the potential to reduce aflatoxin absorption because some LAB cell wall components aid in the binding and removal of aflatoxin from solution. As a result, aflatoxins are less accessible and are removed from probiotic cell walls more quickly. Toxin elimination must occur within living cells, because mycotoxins can be eliminated by microbes and their metabolites^20^. The bacteria colonized within regular fermented yogurt were isolated and identified as capable probiotic microorganisms in this study. The purpose of this research is to investigate how effective two *Lactobacillus* probiotic bacteria are at suppressing aflatoxin production.

## Materials and methods

### LAB strains and culture condition

Fresh traditional fermented yogurt samples from different parts of Iran were collected. The samples were brought on ice to the laboratory of food biotechnology at the Agricultural Biotechnology Research Institute of Iran (ABRII), Tabriz, Iran. To separate the LAB from the sample, inoculate 50 mL of de Man Rogosa and Sharpe broth (MRS, Merck, Darmstadt, Germany) with 25 g/L NaCl and incubate at 37 °C for 48 h. After the incubation period, all cultures were separately drawn on MRS agar plates and anaerobically incubated at 37 °C for 48 h in an anaerobic vessel equipped with an anaerobic gas generation kit. Different forms of colonies were isolated from cultured MRS agar plates. After morphological and biochemical analysis (Gram stain, catalase activity, cell morphology, and spore formation), gram-positive and catalase-negative bacilli and cocci were identified as LAB isolates.

### Acid and bile resistance

To test acid resistance, MRS broth with pepsin enzyme (3 mg/mL) was applied as a medium. With 1.0 N HCl, the pH of the broth was changed to 2.5, and broth (pH 7.0) was utilized as a control. In addition, the broth was inoculated for 3 h to determine the optical density (OD) at 600 nm. Resistance to low pH was calculated by the following formula:

$${\text{Survival rate }}\left( \% \right): \, \left[ {{\text{OD }}\left( {\text{after treatment}} \right)/{\text{OD }}\left( {\text{before treatment}} \right)} \right] \, \times { 1}00\% .$$

Strains with survival rates of more than 80% were chosen for further study^21^. The growth potential of the isolate was tested in the presence of 0.3% w/v bile salts. The samples were inoculated for 4 h at 37 °C, and the OD_620_ of the samples was evaluated to ensure cell viability. The resistivity of oxgall was calculated using the equation below^21^:

$${\text{Survival rate }}\left( \% \right): \, \left[ {{\text{OD }}\left( {\text{After treatment}} \right)/{\text{OD }}\left( {\text{Before treatment}} \right)} \right] \, \times { 1}00\% .$$

### Inhibition of aflatoxin-producing aspergilli by LAB isolates

The modified overlay method was used to assess the inhibition of aflatoxin-producing *Aspergillus flavus* and *Aspergillus niger*^22^ by the two selected LAB isolates. To accomplish this, selected LAB were streaked on MRS agar plates and incubated in an anaerobic container at 37 °C for 48 h. The soft potato dextrose agar (PDA, 75 wt%) was then prepared and overlaid on the MRS plates with fungus spores of defined inoculation size (9.5 × 10^4^ spores/mL) as determined by counting on the Neubauer hemocytometer. The plates were then incubated aerobically at 30 °C for 7 days. Each LAB isolate was tested twice against each *Aspergillus* strain, with one control for each. They were looking for discrete zones of inhibition surrounding the bacterial streaks, which they called “positive impact zones”.

### Antimicrobial activity

An agar well-diffusion experiment was used to determine the antibacterial properties of isolates. For this purpose, Mueller–Hinton agar was used as a medium and some different indicator bacteria, including *Escherichia coli* (PTCC 1276), *Salmonella enterica* (PTCC-1709), *Listeria monocytogenes* (PTCC 1163), *Staphylococcus aureus* (PTCC 1764), *Bacillus cereus* (PTCC 1539), *Streptococcus iniae* (PTCC 1887), *Shigella flexneri* (ATCC 9199), *Klebsiella pneumoniae* (ATCC 43816), and *Yersinia enterocolitica* (ATCC 23715) were used. To make wells in the medium, a sterile, pasteurized pipette was utilized.

Finally, 100 mL of isolate supernatants were poured into each well, and the plates were incubated at 37 °C for 24 h. After the incubation period, the measured inhibitory halo zone diameters were statistically examined^23^.

### Antibiotic susceptibility

The disc diffusion method was used to test isolates susceptibility to many high-consumption and therapeutically significant antibiotics (see Table 1). The isolates were swabbed on solidified MRS medium, antibiotic discs were placed on the medium, and it was incubated overnight at 37 °C. Finally, the size of the suppression zone around the disc was measured with a digital caliper^24^.

**Table 1:** Antibiotic susceptibility of selected strains against the high consumption antibiotics performed by disk diffusion assay.

Strains	Antibiotic susceptibility (Clear zone (mm))														
	S	V	CFM	FEB	K	SXT	TE	CP	AM	E	CC	GM	C	CN	CRO
*ABRIIFBI-6*	19 (S)	11 (R)	21 (S)	23 (S)	5 (R)	18 (S)	20 (S)	15 (I)	22 (S)	29 (S)	16 (I)	12 (S)	18 (S)	21 (S)	24 (S)
*ABRIIFBI-7*	22 (S)	10 (R)	24 (S)	21 (S)	3 (R)	21 (S)	28 (S)	14 (I)	19 (S)	26 (S)	17 (I)	11 (S)	20 (S)	22 (S)	21 (S)

*S* streptomycin, *V* vancomycin, *CFM* cefixime, *FEB* cefepime, *K* kanamycin, *SXT* sulfamethoxazole, *TE* tetracycline, *CP* ciprofloxacin, *AM* ampicillin, *E* erythromycin, *CC* clindamycin, *GM* gentamycin, *C* chloramphenicol, *CN* cephalexin, *CRO* ceftriaxon.

Erythromycin results based on R ≤ 13 mm; I: 13–23 mm; S ≥ 23 mm.

Gentamycin results based on R ≤ 6 mm; I: 7–9 mm; S ≥ 10 mm.

Vancomycin results based on R ≤ 12 mm; I: 12–13 mm; S ≥ 13 mm.

I: intermediate (zone diameter, 12.5–17.4 mm); R: resistant (zone diameter, ≤ 12.4 mm); S: susceptible (zone diameter, ≥ 17.5).

### Cell surface hydrophobicity

As previously mentioned, isolates’ capacity to adhere to xylene and toluene was tested^24^. The The test was run three times and was expressed by the following formula:

$${\text{Hydrophobicity }}\left( \% \right) \, = \, \left( {{1 } - {\text{ A}}_{{1}} /{\text{A}}_{0} } \right) \, \times { 1}00,$$

where A_0_ is the absorbance at 600 nm before xylene and toluene are added and A_1_ is the absorbance after xylene and toluene have been added for 4 h.

### Autoaggregation assay

The ability of isolates to adhere to xylene and toluene was examined, as previously reported. The percentage of auto-aggregation was measured using this formula:

$${\text{Automatic aggregation }}\left( \% \right) \, = { 1 } - \, \left( {{\text{A}}_{{\text{t}}} /{\text{A}}_{0} } \right) \, \times { 1}00,$$

where A_0_ represents the absorbance at time t = 0, and at represents the absorbance at time t.

### Coaggregation assay

Based on the method used, isolates were aggregated against *S. aureus* and *B. cereus*^25^. The coaggregation rate was calculated based on the following formula:

$${\text{A}}_{0} {-}{\text{ A}}_{{\text{t}}} /{\text{A}}_{{\text{t}}} \times { 1}00.$$

### Biofilm formation

The ability of isolates to produce biofilms was assessed using Gomez et al., method ^26^, with some modifications. Sterile 6-well tissue culture plates were filled with 5 mL of MRS broth supplemented with 500 mL of overnight isolate (10^7^–10^8^ CFU/mL). For this purpose, the cultures were anaerobically incubated at 37 °C for 48 h. After this, the wells were gently washed three times with 5 mL of sterile distilled water. The adherent bacteria were then fixed using 3 mL of methanol for 15 min, after which the plates were emptied and dried at room temperature. Then 3 mL of a 2% (v/v) crystal violet solution was poured into the wells and left at room temperature for 5 min. Finally, 2 mL of 33% (v/v) glacial acetic acid was used to lyse the dye from the adherent cells, and the OD of each well was measured at 595 nm with a plate reader (Bio-Rad, Hercules, CA, USA)^26^.

### Molecular identification

#### Genomic DNA extraction

Total genomic DNA was isolated from the isolates using a technique developed in our lab. A single colony of each isolate was inserted in a 0.2 PCR tube, and 20 L of lysis buffer was added to the tube for this purpose. The tubes were vortexed gently until the contents were homogenous, and then left at room temperature for 1 h. They were then incubated for 10 min at 85 °C in a thermal cycler PTC 200 (MJC Research, Waltham, USA). After this time, the tubes were filled with 150 L of deionized water and centrifuged at 8000×*g* for 5 min. Finally, the upper phase containing genomic DNA was taken out of the tubes and poured into new ones. The genomic DNA was kept in the refrigerator until it was needed^27^.

#### Amplification of 16S-rRNA gene by polymerase chain reaction (PCR)

Using LAB-specific universal primers, genomic DNA samples from the isolates were amplified in a PTC 200 thermal cycler^23^. The following temperature profile was used for amplification. First, DNA denaturation at 95 °C for 5 min is followed by denaturation at 94 °C for 60 s, annealing at 59 °C for 60 s, elongation at 72 °C for 60 s, and 32 cycles of the final elongation step at 72 °C for 5 min. PCR products were separated using electrophoresis on a 0.8% (w/v) agarose gel and stained with ethidium bromide.

#### 16S-rRNA gene sequencing

The 16S rRNA gene (1544 bp) PCR products were produced using the aforementioned primer set. Macrogene Corporation, based in South Korea, sequenced the PCR products. The sequences were then examined using the National Center for Biotechnology Information’s BLAST program. (http://www.ncbi.nlm.nih.gov/BLAST).

### Statistical analysis

To determine the significant differences between the parameters of each isolate (P ≤ 0.05), analysis of variance (ANOVA) and Duncan’s test were used. Moreover, Excel 2013 (Microsoft Corporation) and SPSS (IBM SPSS Statistics 20) were used for formal statistical analysis.

## Results

### Morphological and biochemical assays

A total of 31 rod- and cocci-shaped colonies were produced on culture media and identified as LAB isolates using gram-positive and catalase-negative tests. For acid and bile resistance testing, each colony was propagated independently.

### Acid and bile tolerance

After 3 h of incubation at pH 2.5 and 4 h of incubation in 0.3% oxgall, the survival rates of the tested strains varied from 9.44 to 100% and 16.08 to 100%, respectively. According to the findings, most isolates survived between 29 and 63 percent after 3 h of incubation at pH 2.5 and 4 h of incubation in 0.3% oxgall, respectively. Only two isolates (ABRIIFBI-6 and ABRIIFBI-7) demonstrated more than 90% acid and bile resistance, with survival rates of 91 and 96% at pH 2.5 and 92 and 98% in 0.3% bile oxgall, respectively. As a result, these two isolates were chosen for further investigation.

### Antifungal activity

**Figure 1:**
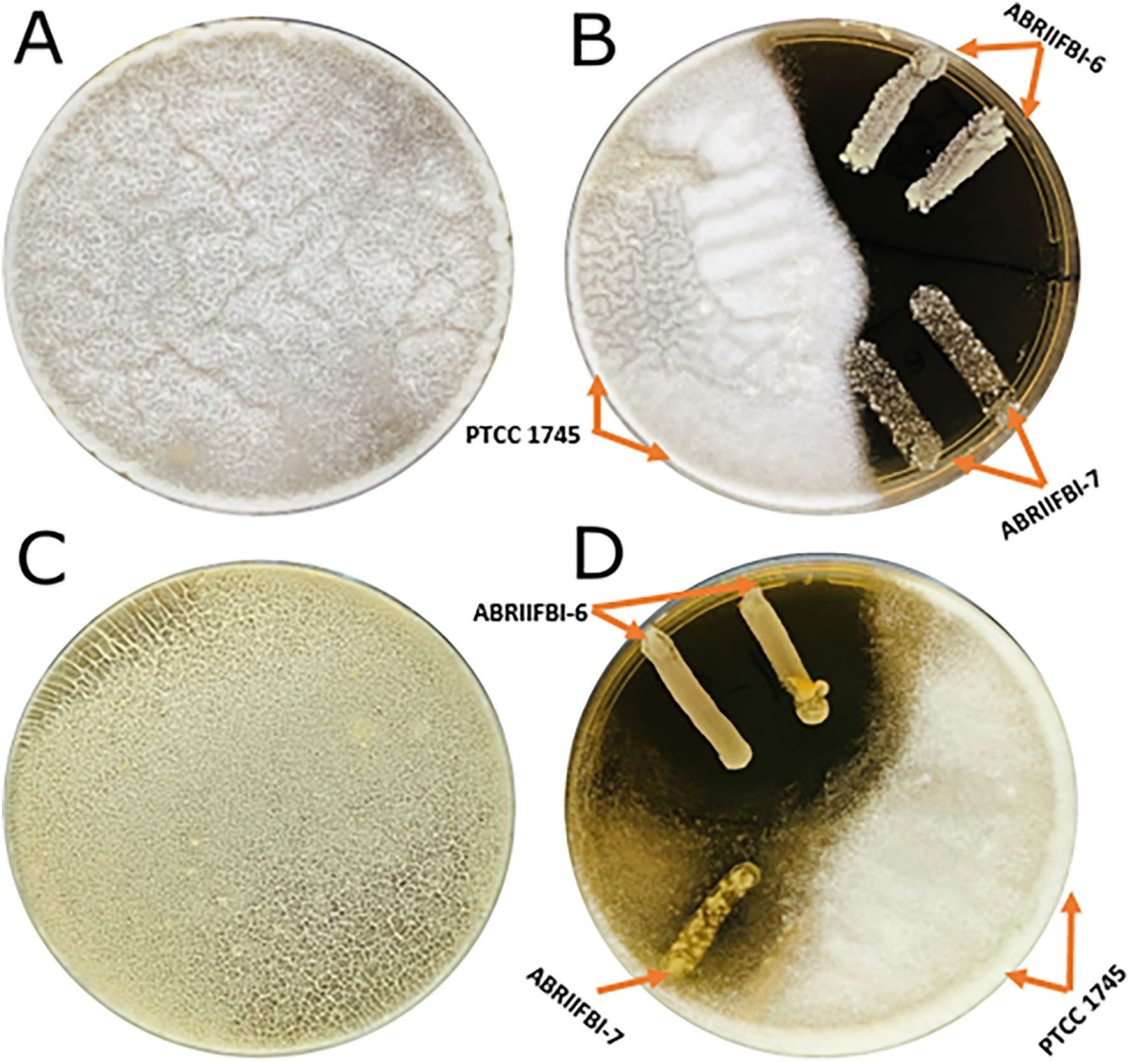
Antifungal activity of potential probiotic strains against *Aspergillus flavus* and *Aspergillus niger* is shown in Figure. (**A**) *A. flavus* covered the entire plate in the absence of probiotics. (**B**) The potential probiotics ABRIIFBI-6 and ABRIIFBI-7 were able to inhibit the growth of *A. flavus*, whereas PTCC 1745, used as a control, was unable to do so. (**C**) *A. niger* covered the entire plate in the absence of probiotics. (**D**) The potential probiotic ABRIIFBI-6 was able to inhibit the growth of *A. niger* similarly to *A. flavus*, but ABRIIFBI-7 only slightly inhibit the growth of *A. niger*. Furthermore, strain PTCC 1745, used as a control, was unable to inhibit the growth of both aspergilli.

The antifungal activity results are shown in Fig. 1. According to the findings, both *A. niger* and *A. flavus* covered the entire plate in the absence of probiotics. The potential probiotics ABRIIFBI-6 and ABRIIFBI-7 were able to inhibit the growth of *A. flavus*, whereas PTCC 1745, used as a control, was unable to do so. The potential probiotic ABRIIFBI-6 was able to inhibit the growth of *A. niger* similarly to *A. flavus*, but ABRIIFBI-7 only slightly inhibits the growth of *A. niger*. Furthermore, strain PTCC-1745, used as a control, could not inhibit the growth of both aspergilli.

### Antimicrobial activity

The antibacterial activity of the tested strains against a variety of gram-positive and gram-negative bacteria is listed in Table 2. Based on results, both strains showed a broad spectrum of antagonistic activity against gram-positive indicator bacteria, including *Listeria monocytogenes*, *Staphylococcus aureus*, *Bacillus cereus*, and *Streptococcus iniae,* whereas they had no effect on gram-negative bacteria, such as *Klebsiella pneumoniae*, *Escherichia coli*, *Yersinia enterocolitica*, *Shigella flexneri*, and *Salmonella enterica*. The maximum zones of inhibition of ABRIIFBI-6 and ABRIIFBI-7 strains were observed for *Streptococcus iniae* (26.3 ± 0.6 mm and 25.5 ± 0.8 mm, respectively).

**Table 2:** Antimicrobial activity of isolates against the pathogenic bacteria.

Strain	Antimicrobial activity (clear zone (mm))								
	*Escherichia coli*	*Salmonella enterica*	*Listeria monocytogenes*	*Staphylococcus aureus*	*Bacillus cereus*	*Streptococcus iniae*	*Shigella flexneri*	*Klebsiella pneumoniae*	*Yersinia enterocolitica*
*ABRIIFBI-6*	0	0	21.9 ± 0.6^ab^	24.2 ± 0.1^a^	23.7 ± 0.9^a^	26.3 ± 0.6^a^	0	0	0
*ABRIIFBI-7*	0	0	22.2 ± 0.5^a^	17.7 ± 0.7^bc^	22.8 ± 0.8^a^	25.5 ± 0.8^a^	0	0	0

Values are mean ± standard deviation. Different lowercase superscript letters are significantly different (P ≤ 0.05). All experiments were repeated twice.

(Strong ≥ 20 mm), (Moderate < 20 mm > 10 mm), and (Weak ≤ 10 mm).

### Antibiotic susceptibility

The tested antibiotics were cefepime (30 g), streptomycin (10 g), sulfamethoxazole (2 g), cefixime (5 g), tetracycline (30 g), kanamycin (30 g), ampicillin (10 g), erythromycin (15 g), clindamycin (2 g), gentamycin (10 g), chloramphenicol (30 g), Both strains were sensitive to most of the antibiotics that were tested, but they were resistant to vancomycin, intermediate to clindamycin, and intermediate to ciprofloxacin, as shown in Table 1.

### Cell surface hydrophobicity

The cell surface hydrophobicity percentages were 61.2 ± 1.4 and 57.3 ± 1.6% for ABRIIFBI-6 and 55.4 ± 1.8 and 51.2 ± 1.4% for ABRIIFBI-7 with toluene and xylene, respectively (Table 3).

**Table 3:** Molecular name, accession numbers, hydrophobicity, autoaggregation, and coaggregation ability of evaluated strains.

LAB strains	Accession numbers	Hydrophobicity (%)		Autoaggregation (%)	Coaggregation (%)	
		With toluene	With xylene		*St. aureus*	*B. cereus*
*Limosilactobacillus fermentum ABRIIFBI-6*	OM666657	61.2 ± 1.4^b^	57.3 ± 1.6^a^	67.4 ± 1.9^bc^	51.3 ± 1.7^a^	57.9 ± 2.1^a^
*Limosilactobacillus fermentum ABRIIFBI-7*	OM698829	55.4 ± 1.8^a^	51.2 ± 1.4^bc^	75.8 ± 2.3^a^	52.4 ± 1.8^a^	49.3 ± 1.9^b^

Values are mean ± standard deviation. Different lowercase superscript letters are significantly different (P ≤ 0.05). All experiments were repeated twice.

### Autoaggregation assay

The autoaggregation capacity of both selected strains is shown in Table 3. The values of autoaggregation ability of strains ABRIIFBI-6 and ABRIIFBI-7 were 67.4 ± 1.9 and 75.8 ± 2.3%, respectively, after 4 h of incubation.

### Coaggregation assay

The findings of the coaggregation ability of both tested strains are shown in Table 3. The coaggregation percentages of strain ABRIIFBI-6 were 51.3 ± 1.7 and 57.9 ± 2.1% with *S. aureus* and *B. cereus*, respectively, whereas the coaggregation percentages of strain ABRIIFBI-7 were 52.4 ± 1.8 and 49.3 ± 1.9% with *S. aureus* and *B. cereus*.

### Biofilm formation

Both strains were able to create biofilms, according to the results. Strain ABRIIFBI-6 showed the strongest biofilm formation value, with OD_595_ = 1.86, whereas strain ABRIIFBI-7 showed the weakest biofilm formation value, with OD_595_ = 0.19.

### Molecular identification

The phenotypic characterization of the selected LAB strains was validated using 16S rRNA gene sequencing. Amplification of the 16S rRNA genes confirmed that both strains belonged to the genus *Limosilactobacillus fermentum*. They were submitted to the NCBI GeneBank with accession numbers OM666657 and OM698829 for ABRIIFBI-6 and ABRIIFBI-7, respectively.

## Discussion

The current investigation clearly reveals that ABRIIFBI-6 and ABRIIFBI-7 strains have an antifungal impact on aflatoxigenic fungal isolates. Nonetheless, ABRIIFBI-6 was more effective than ABRIIFBI-7 in preventing the development of aflatoxin-producing aspergilli strains in vitro. The current study’s findings coincide with those of other researchers that tested Lactobacillus species comparable to those employed in this study but with other LAB strains in the in vitro growth control of Aspergillus spp. and other fungal strains^28^. Lactobacillus strains may limit fungal growth rate by producing secondary metabolites such as organic acids, bacteriocins, and hydrogen peroxide^29^.

It has been shown that selecting suitable potential probiotic strains that are resistant to bile salts and have a high tolerance for acidic conditions is critical in order to colonize the upper gut more effectively^24,30^. In our study, we isolated and identified two promising potential probiotic strains, ABRIIFBI-6 and ABRIIFBI-7, and showed that they were capable of growing at a low acidic pH and surviving in the presence of 0.3% bile oxgall bile in our experiment.

The adhesion qualities of lactobacilli strains have been linked to cell surface hydrophobicity, autoaggregation, and coaggregation and have been demonstrated to be necessary for the protection and colonization of the alimentary canal. A minimum hydrophobicity of 40% is a crucial criterion for potential probiotic strains^31,32^. In this line, our results showed a high hydrophobicity of the selected strains toward toluene and xylene. They also had substantial autoaggregation (ABRIIFBI-6: 67.4 ± 1.9 and ABRIIFBI-7: 75.8 ± 2.3%), indicating that these two dairy strains could probably be appropriate for both animal and human consumption.

Because of safety concerns, the acquired isolates were further examined for antibiotic resistance capabilities. Antibiotics are used as a major tool by the medical industry to combat diverse diseases. However, many pathogens are resistant to antibiotics and present significant danger to the treatment of nosocomial and community-acquired infections^33^. Thus, determining the antibiotic resistance of *Lactobacillus* strains is a critical criterion for selecting suitable potential probiotics, as commercially available probiotics with antibiotic-resistant genes may be passed on to pathogens in the intestine. In our study, we tested the security of ABRIIFBI-6 and ABRIIFBI-7 strains by determining their susceptibilities to fifteen antibiotics. Our results showed resistance of the two selected strains to vancomycin and kanamycin but not to the other antibiotics. This is consistent with previous research that found many *Lactobacillus* species to have high natural resistance to vancomycin and kanamycin^33–35^_._

Bacterial biofilms are an important part of understanding how bacteria adapt to stress and colonize diverse environments. We discovered that the two tested dairy strains (ABRIIFBI-6 and ABRIIFBI-7) have the ability to form biofilms on the bottom of plates. The antimicrobial activity of LAB has been described by Tulumoglu et al.^36^.

Our results demonstrated the antifungal properties of ABRIIFBI-6 and ABRIIFBI-7 strains on aflatoxigenic fungal strains. The potential probiotics ABRIIFBI-6 and ABRIIFBI-7 were able to inhibit the growth of *A. flavus*, whereas PTCC 1745, used as a control, was unable to do so. The potential probiotic ABRIIFBI-6 was able to inhibit the growth of *A. niger* better than ABRIIFBI-7 which only slightly inhibit the growth of *A. niger*. Based on this finding, it could be suggested that ABRIIFBI-6 was the most effective strain in suppressing the growth of *Aspergilli *in vitro*.* Antifungal activity of strains inhibited both *Aspergillus parasiticus* expansion and thus the formation of aflatoxins^36^. *Lactobacillus plantarum, Lactobacillus fermentum*, *Lactobacillus brevis*, and *Lactococcus spp*. have in vitro antifungal activities on aflatoxigenic fungal isolates in proportions similar to those seen in this study. Our findings are consistent with those of Magnusson et al., who investigated *Lactobacillus* species similar to ours but with different LAB strains and controlled the growth of Aspergillus spp. and other fungal strains in vitro^37,38^.

We showed that the effect of the probiotic bacteria suspensions used in this study is extensive, each of them being active against a different *Aspergillus* spp. The formation of secondary metabolites by *Lactobacillus* strains on fungal species could possibly be the reason for their inhibition of expansion rates. Lactic and acetic acids are the most common results of LAB’s carbohydrate fermentation. These acids pass through the membranes of target species in their undissociated hydrophobic state, lowering cytoplasmic pH and inducing cell death^38,39^. Despite the lack of compelling evidence that protein molecules play a role in growth inhibition, several authors discovered that several carboxylic acid strains developed antifungal metabolites that were sensitive to proteolytic enzymes^22^.

In summary, our study demonstrated that strains ABRIIFBI-6 and ABRIIFBI-7 could be used as potential probiotics. We found that these selected strains could have potent antibacterial properties against gram-positive bacteria and two important species of *Aspergillus*. So, they could be used as biocontrols for testing in vivo to find out what health benefits they might have and as new probiotic strains in the food industry.

## Data Availability

The datasets generated and/or analysed during the current study are available in the NCBI GeneBank repository, Accession Numbers OM666657 and OM698829.
